# **S**irtuins and Their Roles in Brain Aging and Neurodegenerative Disorders

**DOI:** 10.1007/s11064-016-2110-y

**Published:** 2016-11-24

**Authors:** Henryk Jęśko, Przemysław Wencel, Robert P. Strosznajder, Joanna B. Strosznajder

**Affiliations:** 1 Department of Cellular Signalling, Mossakowski Medical Research Centre, Polish Academy of Sciences, 5 Pawińskiego st., 02106 Warsaw, Poland; 2 Laboratory of Preclinical Research and Environmental Agents, Department of Neurosurgery, Mossakowski Medical Research Centre, Polish Academy of Sciences, 5 Pawińskiego st., 02106 Warsaw, Poland

**Keywords:** Sirtuins, Brain aging, Alzheimer’s disease, Parkinson’s disease, Neuroprotection, Transcription factors

## Abstract

Sirtuins (SIRT1–SIRT7) are unique histone deacetylases (HDACs) whose activity depends on NAD^+^ levels and thus on the cellular metabolic status. SIRTs regulate energy metabolism and mitochondrial function. They orchestrate the stress response and damage repair. Through these functions sirtuins modulate the course of aging and affect neurodegenerative diseases. SIRTSs interact with multiple signaling proteins, transcription factors (TFs) and poly(ADP-ribose) polymerases (PARPs) another class of NAD^+^-dependent post-translational protein modifiers. The cross-talk between SIRTs TFs and PARPs is a highly promising research target in a number of brain pathologies. This review describes updated results on sirtuins in brain aging/neurodegeneration. It focuses on SIRT1 but also on the roles of mitochondrial SIRTs (SIRT3, 4, 5) and on SIRT6 and SIRT2 localized in the nucleus and in cytosol, respectively. The involvement of SIRTs in regulation of insulin-like growth factor signaling in the brain during aging and in Alzheimer’s disease was also focused. Moreover, we analyze the mechanism(s) and potential significance of interactions between SIRTs and several TFs in the regulation of cell survival and death. A critical view is given on the application of SIRT activators/modulators in therapy of neurodegenerative diseases.

## Introduction

Sirtuins (SIRT1–SIRT7) belong to the family of histone deacetylases. These enzymes modulate the properties and functions of proteins (e.g. histones, kinases, and transcription factors-TFs) [1, 2] by removing acetyl groups post-translationally attached to their lysine residues by acetyltransferases. Sirtuins are class III HDACs and differ from other classes in that they require NAD^+^ for their activity. This feature couples sirtuin activity to the cellular metabolic status [3], in turn allowing these enzymes to modulate the crucial proteins of the electron transport chain (ETC), stress response, and life/death signaling. Some sirtuins also possess additional enzymatic activities such as mono(ADP-ribosyl)ation (SIRT3, SIRT4, SIRT6), the ability to remove a wide array of other lysine modifications (e.g. desuccinylation and demalonylation—SIRT5; decrotonylation—SIRT1–3), and/or lack detectable deacetylation capability (SIRT4) [1, 2]. Sirtuins are engaged in cross-talk with a wide spectrum of transcription factors, including forkhead box subgroup O (FOXOs), p53, and NF-κB, and with proteins engaged in DNA repair such as DNA-dependent protein kinase (DNA-PK) [1, 4]. The versatile and ubiquitous family of poly(ADP-ribose) polymerases (PARPs) shares the feature of NAD^+^-dependence with sirtuins; the two classes of enzymes compete for the substrate and interact in numerous ways, influencing a very broad range of cellular functions [1, 4]. Sirtuins display complex cellular localization in the cytoplasm, nucleus, and mitochondria [2]. All sirtuins are present in the brain in a highly regulated, spatiotemporal pattern and may influence the course of aging and pathological changes [4, 5].

## Sirtuins and Their Roles in Mitochondria: Biogenesis, Energy Production, and Survival/Death Signaling

**Fig. 1:**
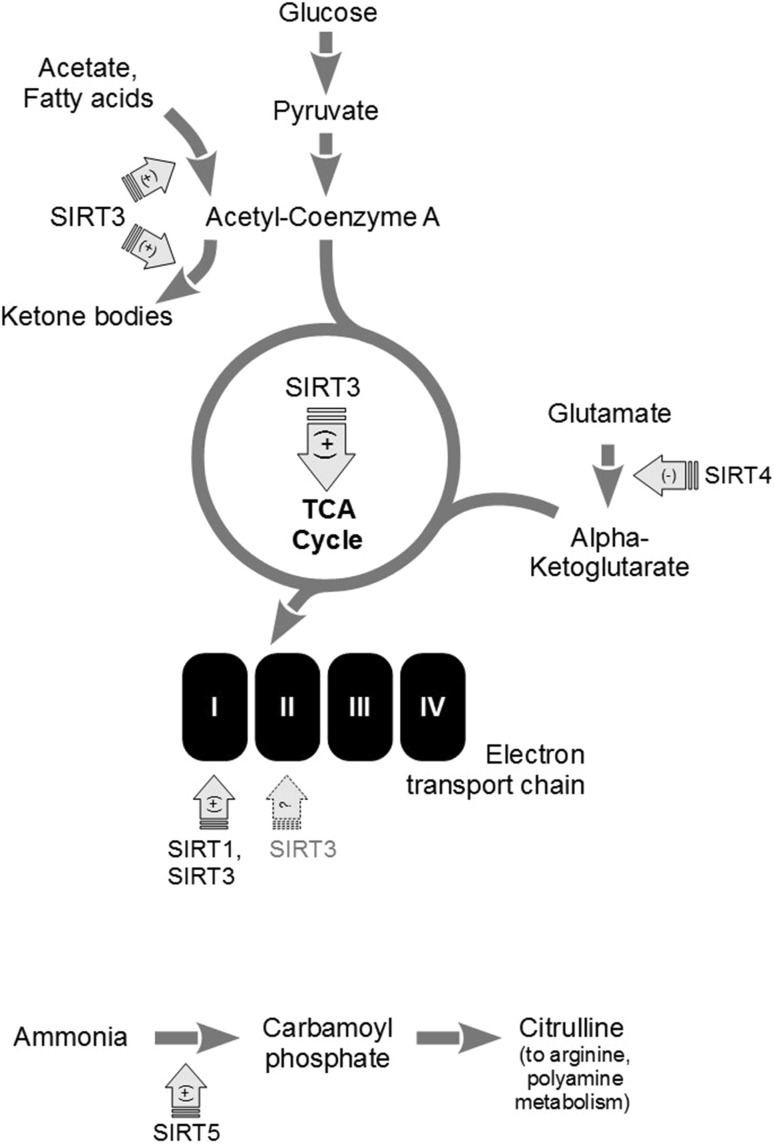
Mitochondrial targets of sirtuin signalling. Depending on the organ and cell type, sirtuins may affect multiple stages of glucose-based energy metabolism, the production of ketone bodies, glutamate usage, or arginine, citrulline, and polyamine biosynthesis. While numerous metabolites have direct roles in the CNS, others not produced locally, may dramatically impact brain health (as in the case of e.g. urea cycle, which is typically incomplete in the central neurons, but its deregulation in peripheral tissues leads to neurodegeneration in the CNS). According to [40], modified

The presence of sirtuins (SIRT3, 4, 5) in mitochondria appears to undergo precise regulation. The exact localization of SIRT3 seems to be species-specific; human SIRT3 is a mitochondrial matrix protein, but its mouse ortholog resides in the inner membrane [6, 7]. SIRT4 and SIRT-5 are also present in the mitochondrial matrix. Human SIRT5 has an additional membrane insertion sequence; its mitochondrial presence depends on the isoform [8]. Mitochondrial localization of sirtuins is mutually interdependent. It is proposed that SIRT3 is present in mitochondria only when the expression of SIRT5 is low [9]. This scattered evidence suggests the possibility of a complex network of regulation for the level and localization of various sirtuins. The results published thus far point to the involvement of sirtuins in the regulation of mitochondrial turnover, fusion and fission, and of mitochondrial cell death signaling. Sirtuins also influence mitochondrial respiratory machinery and ROS production in multiple tissues (Fig. 1). Importantly, the significance of mitochondrial regulation for CNS homeostasis extends well beyond brain neurons, as they are extremely sensitive to the effects of metabolic deregulation in the periphery (with the arginine/urea metabolism being an example of a sirtuin-dependent pathway strongly linked to neurodegenerative conditions). SIRT3 can also enhance via FOXO3 the expression of antioxidant enzymes including the mitochondrial manganese superoxide dismutase (Mn-SOD), peroxiredoxins, or thioredoxin 2 [10, 11].

SIRT1, mainly a nuclear enzyme, can be also present in mitochondria [12]. Moreover, it has been shown to be engaged in mitochondrial biogenesis [13–15]; reviewed in [12, 16]. Exercise training increases SIRT1 mRNA level and the amount of mtDNA indicating intensified mitogenesis in most brain regions, with potential cognitive significance [15]. SIRT1 seems to exert this beneficial influence via peroxisome proliferator-activated receptor γ co-activator-1α (PGC-1α) [17]. PGC-1α is a crucial regulator of mitochondrial biogenesis and energy metabolism [18, 19]. PGC-1α is also engaged in antioxidant defense, for example via regulation of Mn-SOD and glutathione peroxidase [20]. Impaired PGC-1α function may contribute to the pathogenesis of neurodegenerative diseases such as Alzheimer’s and Parkinson’s (AD and PD, respectively), Huntington’s disease, or ischemic damage [21–24]. SIRT3 is also involved in the regulation of mitochondrial biogenesis in a manner mediated by its target FOXO3 and Parkin. SIRT3 activates FOXO3a and its target PTEN-induced kinase-1 (PINK-1), a protein known to modulate the cellular redox status and mitochondrial function. PINK-1 in turn enhances Parkin activity, potentiating the fusion of mitochondria and mitophagy [25]. SIRT3 overexpression has led to a significant increase in cellular mtDNA content, while shRNA against SIRT3 has reduced the PGC-1α-mediated rise of mtDNA [26].

The influence of sirtuins on the energy metabolism may also come from their direct interactions with the respiratory machinery (Fig. 1). SIRT3 regulates pyruvate dehydrogenase that is acetylated by the acetyl-CoA acetyltransferase 1 (ACAT1); acetylation/deacetylation status of the dehydrogenase is important for the regulation of glycolysis in cancer cells [27]. Moreover, SIRT3 deacetylates and stimulates isocitrate dehydrogenase 2, an enzyme of the tricarboxylic acid cycle [28]. Complex I constituent, NADH dehydrogenase 1α subcomplex subunit 9 is deacetylated and activated by SIRT3 [29]. SIRT1 has been shown to enhance the function of complex I in insulin-resistant cells, possibly via SIRT3. Overexpression of SIRT1 attenuated high-fat diet-induced insulin resistance in the skeletal muscle, and restored the levels of SIRT3, mitochondrial antioxidant enzymes and DNA [30]. The part of complex II, succinate dehydrogenase subunit A is also suggested as a SIRT3 substrate [31]. Thus, sirtuins appear to influence several stages of energy metabolism. SIRT4 generally falls in the same scenario. Loss of its expression in several cell types (hepatocytes, muscle) leads to lower ATP production. SIRT4 has been implicated in the regulation of mitochondrial uncoupling. It is also involved in signaling to the nucleus via AMPK, PGC1α and acetyl-CoA carboxylase, which adjusts mitochondrial ATP production to the energetic demands of the cell [32]. SIRT5, too, has been found to be linked to AMPK, PGC1α and mitochondrial ATP generation [33].

Mitochondrial sirtuins are involved in the usage of alternative energy sources. The change of energetic substrates is accomplished in hepatocytes by SIRT3 through deacetylation of acyl-CoA dehydrogenases, glutamate dehydrogenase, and the mitochondrial acetyl-CoA synthetase [34–36]. These activities allow sustained energy production in the conditions of disturbed supply of the basal substrates. SIRT4 has been found to shift the balance in lipid usage from fatty acid oxidation towards lipid anabolism, by inhibiting malonyl-CoA decarboxylase [37]. Mitochondrial lipid metabolism can be also affected by SIRT5 via its desuccinylase activity directed towards liver mitochondrial proteins engaged in β-oxidation and ketogenesis [38]. SIRT5 might also influence other aspects of mitochondrial energy production such as the tricarboxylic acid cycle [39].

Besides the ATP generation, SIRT5 regulates the detoxication of ammonia. Through deacetylation, SIRT5 activates the carbamoyl phosphate synthase 1, intensifying the conversion of ammonia into carbamyl phosphate and then citrulline, which is metabolized in the urea cycle (Fig. 1; [40]).

Sirtuins exert their influence on the antioxidative defenses in mitochondria. While PGC-1α is induced by SIRT1 in rat hippocampus [41], Kong et al. [26] have shown that SIRT3 is an important mediator of the PGC-1α-dependent induction of SOD2 and glutathione peroxidase-1 (in skeletal muscle cells). A number of articles confirmed the role of SIRT3 in the positive regulation of the level and activity of MnSOD in various tissues [42–44]. SIRT3 also plays a role in the mitochondrial unfolded protein response, which is activated to cope with oxidatively damaged proteins [45].

Sirtuins have been shown to be involved in the regulation of mitochondrial membrane permeability. In cardiac muscle, SIRT3 deacetylates mitochondrial protein cyclophilin D, which is a regulatory component of the permeability transition pore (mPTP) [46]. SIRT5 deacetylates cytochrome c in vitro [47]. However, the outcome of these phenomena is unclear.

## Sirtuins in Aging

The emerging involvement of sirtuins and their targets in the longevity effects of caloric restriction (CR) may be an excellent recapitulation of their roles in the organism’s struggle to control and counter stress and macromolecular damage [48]. Sirtuins are bi-directionally linked to the signaling pathways of insulin and insulin-like growth factor-I (IGF-I), collectively termed IIS (insulin/IGF signaling). IGF-I increases SIRT1 expression via JNK1 (c-Jun N-terminal kinase 1 [49]). In turn, SIRT1 and SIRT2 restore the activity of the IGF/insulin receptor target Akt, and SIRT1 supports the IIS signal by deacetylation of insulin receptor substrate 2 (IRS-2). However, SIRT1 and SIRT6 could also suppress the expression of IGF, its receptor, and IIS-dependent genes in some circumstances [49]. IIS plays highly regulated, important roles in the CNS. IGF-I synthesis declines in old organisms, weakening IGF’s trophic action and most probably causing a significant proportion of observed age-related disturbances in brain function [50–52].

Despite the generally trophic role of IGF-I the IIS pathway turns out to be a crucial element of longevity-inhibiting signaling [53]. In invertebrate models of aging, IIS-dependent suppression of FOXO ortholog (DAF-16) is relieved in conditions of stress such as oxidative damage, starvation, or CR. This de-repression leads to the activation of DAF-16/FOXO-responsive genes, enhancing the resistance to broad range of stress conditions [54–57].

Data obtained in vertebrates also suggest the involvement of IIS in the modulation of stress resistance and, possibly, longevity [53, 58–60]. The effect was dependent on neuronal action of IIS [61, 62]. However, the matter is still not fully settled suggesting that the much higher complexity and redundancy of IIS in mammals requires far more in-depth analysis [63, 64].Significant side-effects of reduced IIS also complicate the matters [65–67].

**Fig. 2:**
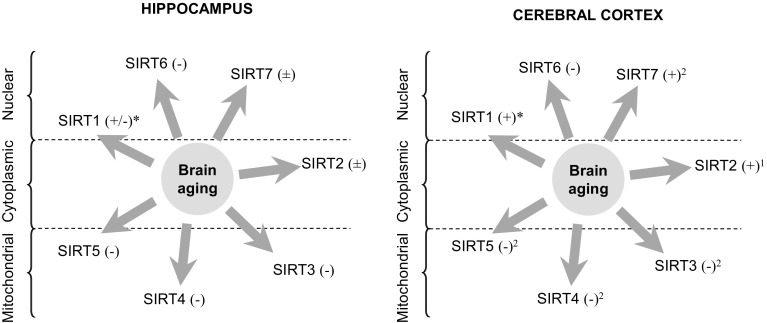
Changes in the protein levels of various sirtuins in the aged rat brain. The influence of physiological brain aging on the protein levels of various sirtuins in the rodent model. 24 months old rats are compared to adults (3 months old). Predominant cellular localizations of sirtuin proteins are marked in hippocampus and cerebral cortex. ^±^No change. *Increased protein but lower activity [71]. ^1^Change observed only in the occipital but not frontal or temporal lobes of the cerebral cortex. ^2^Only in frontal but not occipital or temporal lobe

Sirtuins appear to be involved in the longevity-modulating role of IIS; the impact of SIRT1 on long-term survival occurs again through signaling events in specific regions of the CNS [68]. SIRT1 also appears to be involved in the role of IIS in the CR, but sirtuins might also affect the calorie intake itself—again, through the influence on FOXO [54, 69]. A drop in hippocampal SIRT1 level or activity (Fig. 2) has been noted in the aged rat brain, although the results are inconsistent with some works showing reduced activity despite elevated protein [70, 71].

The expression of SIRT3-SIRT7 undergoes changes in the aging brain in a region-specific manner (Fig. 2; [71, 72]). Single-nucleotide polymorphisms in *SIRT3, SIRT5* and *SIRT6* genes have been noted to correlate with human lifespan [73].

The potential role for SIRT2 in aging is suggested by the association found between human longevity and a polymorphism in the probable regulatory elements of its gene [74]. Isoform-/region-specific increase of brain SIRT2 content has been observed during aging in mice and rats [71, 75]. Deacetylation by SIRT2 of the life-span modulating cell cycle checkpoint kinase BubR1 has been shown to preserve its cellular levels while loss of BubR1 is observed in aging muscle due to NAD^+^ decline [76, 77]. This makes SIRT2 a good candidate for another longevity modulator although it does not seem to be the sole BubR1 regulating factor [78].

SIRT3 single nucleotide polymorphism also seems to associate with human longevity [79, 80], although the data still needs further elaboration [81]. SIRT3 reacts to nutritional status and has been shown to mediate some of the beneficial effects of CR, including many of the CR-induced transcriptional changes in numerous tissues [28, 82, 83]. SIRT3 is increasingly viewed as a modulator of metabolic adaptation to caloric restriction, making it a promising target [84]. Its protein expression changes in a number of mouse peripheral organs during aging, including mouse hematopoietic stem cells where its decrease limits their regenerative potential [85, 86]. Intense oxidative stress reduces SIRT3 in human mesenchymal stromal/stem cells, which renders them more vulnerable as SIRT3 supports the activity of the catalase-SOD ensemble [87, 88]. Disturbances in the SIRT3 role as an important free radical defense supporter also appear to contribute to aging of the central auditory system [89]. Moreover, the repertoire of SIRT3 interacting partners suggest further aspects of its role in longevity. Deacetylation by SIRT3 supports the stability and activity of 8-oxoguanine-DNA glycosylase-1 (OGG1), a base excision DNA repair enzyme. This protects mtDNA against accumulation of the mutagenic damage product 8-oxoguanine [90]. SIRT3 also deacetylates DNA repair regulator Ku70 [91]. In addition, SIRT3 binds the heat shock protein HSP70 and causes its increased nuclear presence [92]. These interactions are potentially linked to the mechanisms of age-related neurodegeneration.

Corresponding with SIRT6 role in glucose metabolism and IGF-I homeostasis, results have been obtained suggesting its involvement in CR [93, 94]. Animal models provide somewhat conflicting results on SIRT6 levels during aging [95–97]; some of the age dependency may be explained by the regulatory loop that links SIRT6 with the age-modulated microRNA miR-766 [98]. The potential engagement of SIRT6 disturbances in the aging process is otherwise among the best documented. Suppression of SIRT6 protein levels mediates premature senescence-like phenotype in cells under H_2_O_2_-induced oxidative stress [99]. Premature cell senescence in Hutchinson–Gilford progeria syndrome (HGPS) and chronic obstructive pulmonary disease is linked with lower SIRT6 expression; its restoration remedies a number of senescence-linked traits, in the latter case through modulation of IIS–mTor signaling [100, 101]. The restoration of falling SIRT6 levels also rescues the diminished efficiency of DNA base excision repair in human foreskin fibroblasts from aged donors [102]. Likewise, in the aged brain diminished SIRT6 binding could lead to genomic instability [103]. In turn, some peripheral tissues display an age-related rise of SIRT6; its inhibition by physical exercise improved oxidative damage resistance in muscle [96]. SIRT6^−/−^ mice develop (possibly IGF-I-linked) progeroid-like phenotype, while SIRT6 overexpression supports male longevity in mice which is accompanied by a reduction in serum IGF-I, dramatic increase in the expression of IGF-binding protein-1 mRNA, and changed phosphorylation levels of Akt and FOXO1 [104, 105]. Moreover, SIRT6 binds c-Jun and inhibits its IGF-dependent transcriptional activity [106]. Analysis of SIRT6 interactions (PARP-1, DNA-PK catalytic subunit, other DNA repair proteins, histones) also supports its role in aging, probably through the regulation of chromatin assembly state to facilitate DNA repair in a way somewhat reminiscent of the role of its partner PARP-1 [107]. SIRT6 localizes early to double-strand DNA breaks and is needed for their efficient removal via both pathways: homologous recombination (HR) and non-homologous end-joining (NHEJ) [108, 109]. The mentioned drop in SIRT6 expression during cellular senescence is accompanied by HR deficiency and SIRT6 overexpression largely rescued this phenotype [110]. Cells deficient in SIRT6 enzymatic activity display defects in base excision DNA repair, increased sensitivity to ionizing radiation (but not UV) and multiple chromosomal aberrations though the results clearly need further elucidation [104]. The links between SIRT6, DNA repair, and aging also extend to telomere maintenance. SIRT6 localizes to telomeric chromatin and facilitates the binding of Werner syndrome (WS) protein (WRN) there. WRN is a DNA helicase crucial for genome stability, mutated in the WS. SIRT6 deficiency leads to replicative senescence and telomere dysfunction resembling the pathology seen in WS [111].

The engagement of SIRT6 in the mitigation of aging and oxidative stress also occurs through its interactions with several crucial pathways of transcriptional regulation. SIRT6 has been found to support the transactivation of anti-oxidant genes by nuclear factor erythroid 2-related factor 2 (NRF-2). SIRT-6 deficiency has led to oxidative stress and accelerated decay of human mesenchymal stem cells [112]. NF-κB, another SIRT6 partner, potentially belongs to the crucial modulators of age-related phenotypes [113]. The interaction of SIRT6 with NF-κB subunit RelA recruits SIRT6 to NF-κB target sequences and allows it to repress promoter activities; many of these belong to a group of genes that show increased expression with age [113, 114]. Experimental SIRT6 deficiency led to hyperacetylation of histones bound to NF-κB target promoters. This increased the activity of these promoters, augmenting NF-κB-dependent cellular senescence. This role of NF-κB has been confirmed in vivo [114]. Hypoxia-inducible factor (HIF) transcription factors are another family of SIRT6 (and SIRT1) interaction partners. The vast significance of HIFs for the regulation of oxygen + glucose/lactate metabolism suggests their engagement of in the course of aging. In invertebrates HIF-related modulation of the lifespan has been shown, though conflicting views exist whether the pathway is separate from CR- and IGF-dependent longevity modulation [115, 116]. The above mentioned data and the shortened lifespan of SIRT6-deficient rodents (accompanied by disturbed glucose metabolism) [117] suggest that SIRT-HIF cross-talk might potentially be also engaged in vertebrate longevity. It is known that SIRT1 can inhibit HIF1 and activate HIF2, and that SIRT6 may be a co-repressor for HIF-1α [117–119]. HIF transactivation targets include genes with known neuroprotective influence, although their role in neurodegeneration is still ambiguous [120, 121].

Sirt7 has been recently noted to support the regenerative potential hematopoietic stem cells via regulation of mitochondrial stress signaling [122]. Its numerous interactions with enzymes of nucleic acid metabolism strengthen the possible association with life-long maintenance, necessitating further research in the topic [107].

The signaling targets of sirtuin-regulated FOXOs with potential anti-aging significance are still rather unclear; candidates include thioredoxin-interacting protein (Txnip), which is repressed by FOXO1a [123]. Txnip1 suppresses the stress response, correlates negatively with longevity and is viewed as a SIRT1 antagonist [124, 125]. FOXOs also target microRNAs that might modulate stress resistance and long-lived dormant invertebrate developmental states [126]. Several other TFs have been suggested as mediators of the pro-longevity SIRT1 action, but their significance needs further elucidation [127].

## Sirtuins in Neurodegeneration and Neuroprotection

### Sirtuins in AD

**Fig. 3:**
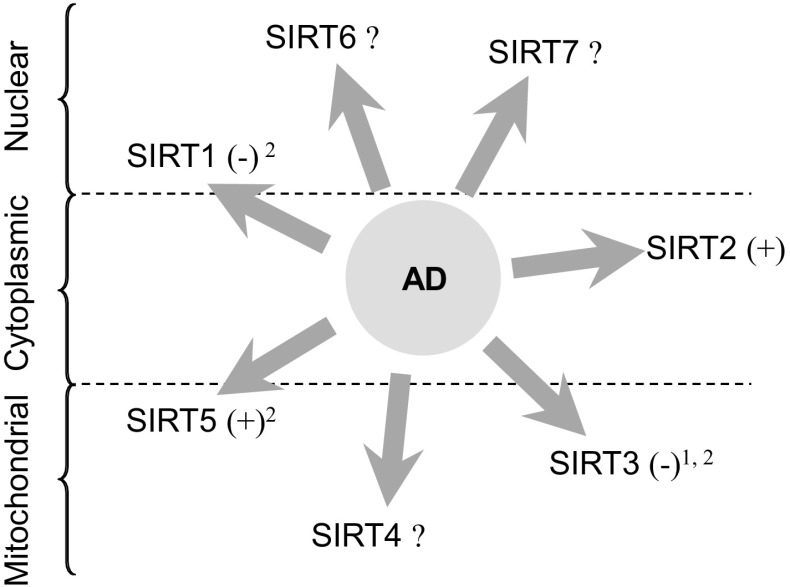
Changes in the levels of various sirtuins in the course of AD. The influence of AD pathology on the expression levels of various sirtuins in the human brain. Predominant cellular localizations of sirtuin proteins are marked. ^±^No change. ^1^Increased mRNA expression observed in the temporal cortex [131]. ^2^Negative (SIRT1, SIRT3) and positive correlation (SIRT5) of immunoreactivity in the hippocampus with Braak neuropathology staging [129]

A number of works have shown the potential role of sirtuins in AD (Fig. 3) and other neurodegenerative disorders. The reduction of SIRT1 and SIRT3 mRNA/protein levels observed in AD brain correlates with the stage/duration of the disease [128, 129], and can be mimcked in vitro by the influence of Aβ_25-35_ on SIRT1 [130]. In turn, up-regulation of *SIRT3* mRNA that followed the spatial and temporal profiles of Aβ accumulation has been shown in mice, and higher *SIRT3* mRNA was observed in the temporal cortex of AD cases (Braak tangle stage III–VI, average age 82.5 ± 2.3) [131]. SIRT5 is induced in activated microglia of AD brains [129]. In vitro Aβ_1-42_ treatment also led to increased SIRT-3, -4, and -5 [132]. However, overexpression of APP and presenilin 1 has led to reduction in SIRT3 mRNA and protein in a mouse model, suggesting more complex relations [133].

**Fig. 4:**
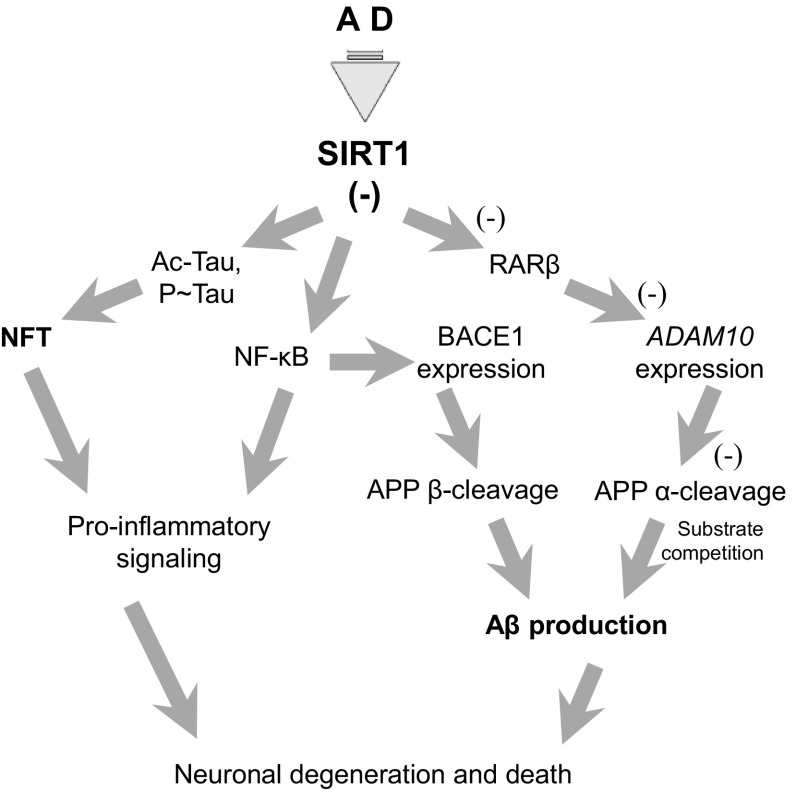
The significance of SIRT1 in Alzheimer’s disease. SIRT1 modulates multiple pathways that adjust the metabolism of Aβ to keep its levels within physiological limits. The sequence of events occurring in AD reduces SIRT1-dependent effects: tau deacetylation, inhibition of the NF-κB pathway, and the α-cleavage of APP, leading to elevaed Aβ and to intensified pro-inflammatory signaling

It has been reported that SIRT1 shifts the balance between amyloidogenic and non-amyloidogenic processing of APP in vitro and in transgenic mouse models [134]. SIRT1 up-regulates the α-secretase ADAM10, and through inhibition of NF-κB down-regulates the expression of the β-secretase β-site AβPP-cleaving enzyme 1 (BACE1) (Fig. 4; [135–141]). Moreover, Aβ degradation via autophagy may also be dependent on SIRT1 [142]. Thus, SIRT1 appears to reduce the levels of Aβ, oxidative stress and the resulting neuronal loss [139]. Activation or overexpression of SIRT1 is also reported to interfere with Aβ toxicity mediated by microglia through its ability to inhibit NF-κB signaling [143, 144, 148]. SIRT1 might also protect against synapse loss, a more subtle and earlier effect of Aβ pathology [139]. In turn, small-molecule SIRT2 inhibitors 3-(1-azepanylsulfonyl)-*N*-(3-bromphenyl) benzamide (AK-7) and 2-cyano-3-[5-(2,5-dichlorophenyl)-2-furanyl]-*N*-5-quinolinyl-2-propenamide (AGK2) have shifted the balance between α- and β-secretase reducing the Aβ load and led to cognitive improvement in two transgenic mouse models [145]. AGK-2 also reduced glial activation by Aβ_1-42_ [144]. Thus, SIRT1 and SIRT2 seem to influence the APP cleavage in approximately opposing ways.

Less data is available for other sirtuins. It has been found that short-term treatment with extracellular Aβ_1-42_ oligomers enhanced the expression of SIRT4 gene but prolonged treatment affected all three mitochondrial isoforms (SIRT3 to SIRT5), suggesting that links between APP/Aβ and SIRTs might be more complex, possibly reciprocal [132].

Intracellular accumulation of pathologically modified microtubule associated protein tau may be another highly promising target in AD research and therapy [146]. Sirtuins mediate the leptin-dependent inhibition of tau phosphorylation [147]. SIRT1also removes acetyl groups from tau, thus relieving the p300-mediated inhibition of phospho-tau degradation [148]. Manipulations of sirtuin activity could therefore influence tau, potentially changing the number of neurofibrillary tangles (NFT) [149, 150]. Moreover, SIRT1 and tau share common upstream regulation mechanism, as both are targets of microRNA-132 [151] and of ademosine monophosphate-activated kinase (AMPK, which leads to the inhibition of the crucial tau kinase GSK-3β, and modulates SIRT1 signaling in a complex manner) [152–154]. These might contribute to the observed inverse correlation between abnormal tau deposition and SIRT1 mRNA and protein levels in AD [128].

Besides Aβ and tau, the two crucial elements of molecular AD pathology, sirtuin signaling is able to influence pathways engaged in neuroprotection and brain tissue renewal. The SIRT1/retinoic acid receptor β target ADAM10 not only cleaves APP but also induces Notch receptor cleavage [155]. The release of Notch intracellular domain activates the transcription of neurogenesis-related genes, and Notch pathway has been shown to be a necessary element of neurogenesis and differentiation of the newly created cells in response to pathological insults [156, 157]. Moreover, Notch targets include genes crucial for synaptic plasticity, learning and memory, and generation of neurites and synapses [155]. Thus, the protection offered by SIRT1 appears to be multi-tiered and stem both from Notch activation and influences on APP and tau metabolism.

A neuroprotective role of SIRT1 has been also observed in prion diseases [158]. Somewhat surprisingly, numerous results point to detrimental roles played by SIRT2 in neurodegenerative disorders, and in other pathological conditions. SIRT2 is increased in AD; its knock-out or inhibition reduces the cytoskeletal pathology and improves autophagy [159]. A meta-analysis has found an association between a polymorphism in an intron of *SIRT2* gene and AD susceptibility [160].

Sirtuin partners FOXOs and the IIS have vast potential significance for AD and other diseases linked to disturbed somatic maintenance. The significance of brain IGF-I signaling and its targets for neuronal survival and death is still poorly known and appears to be fundamentally different from their peripheral roles [161].

IIS has recently become a focus in the research deciphering metabolic disturbances that co-occur with (and possibly precede) AD, raising some hopes for the search of early, measurable symptoms of developing pathology [162]. IIS can suppress Aβ production [163] and resulting tissue damage [164] although its full role in AD is still unclear [165, 166]. Deeper understanding is necessary as it may become an attractive target in the future treatment of AD and PD [167]. However, despite the discrepancies IGF-I replacement therapies have been proposed and tested [161, 168].

FOXOs themselves are capable of extensively modulating protein turnover and oxidative stress, both crucial for Aβ/ASN accumulation and toxicity [169]. FOXOs might also mediate the inhibition of neuroprotective PI3K/Akt signaling by Aβ [170]. These TFs have been thus suggested as potential *integrating factors* in AD metabolic deregulation [171]. The expression of FOXO1 is altered with increased AD severity [172]. FOXO3a might also mediate the toxic effect of Aβ-dependent inhibition of neuroprotective PI3K/Akt signaling [170], and the impact of age on FOXO3 has been suggested as a crucial step changing relatively benign protein aggregates into neurotoxic Aβ deposits [169]. FOXO3a also modulates toxic aggregation of ASN [173] and is found in Lewy bodies/Lewy neurites [174].

### Sirtuins in PD

The course of PD, another neurodegenerative disorder that impacts the dopaminergic system also is affected by SIRT signaling. SIRT1 displays neuroprotective properties in experimental PD models [175, 176]. It was reported that oxyresveratrol protected dopaminergic SH-SY5Y cells against the toxicity of the Parkinsonian mimetic 6-hydroxydopamine through countering the-down regulation of SIRT1. Resveratrol whose functions include activation of SIRT1 also offered protection in this model, as well as in MPTP-induced mouse Parkinsonism [177, 178]. Moreover, genetic variants that result in reduced SIRT1 expression co-occurred with sporadic PD [179].

SIRT1 might exert its protective effects in PD through several pathways linked to general stress resistance and more specifically to α-synuclein (ASN) metabolism. The activation of PGC-1α, a protein considered a central element of oxidative stress resistance, by SIRT1 in response to resveratrol may render MPTP-treated mice less prone to neurodegeneration [180]. The protective effect of resveratrol in a rotenone-induced human neuroblastoma cell model of PD has been largely attributed to its ability to induce autophagic degradation of ASN via SIRT1 [181]. Molecular chaperones may also be valuable targets in protein misfolding-related diseases; Hsp70 has been found to protect against ASN aggregation and toxicity [182, 183]. SIRT1 deacetylated the heat shock factor 1 (HSF1) facilitating prolonged binding to its target sequence in the gene coding for Hsp70. This led to elevated expression of Hsp70 in stress conditions [184] raising the possibility that HSF1 and Hsp70 might indeed mediate the protective effect of SIRT1 as it does for example in an amyotrophic lateral sclerosis model [185].

On the contrary, inhibition of SIRT2 with AK-7 reduces MPTP-induced loss of dopaminergic neurons in a mouse model [186]. SIRT2 inhibition improves neurological and behavioral deficits in a PD model induced by MPTP in old mice [187]. siRNA against SIRT2 or its inhibitor AGK2 block the toxic effect of α-synuclein in a Parkinsonian primary midbrain culture model (mutant ASN transfection) and modifies the pattern of α-synuclein inclusions in cells transfected with ASN and its interaction partner synphilin 1 [188]. SIRT2 inhibition improves neurological and behavioral deficits in a PD model induced by MPTP in old mice [187].

SIRT2 inhibition also blocked the apoptosis of an oligodendroglial cell line in a model of another ASN-linked disorder, multiple system atrophy [189]. Results in cerebral ischemia are less clear [186, 190]. However, SIRT2 has also been shown to contribute to the pathology of the vascular system and to the effects of oxidative stress in the endothelium, which have immediate impact on brain oxygen supply [191, 192].

### Sirtuins in HD

Huntington’s disease (HD) is an autosomal trinucleotide repeat disorder characterized by striatal and cortical neurodegeneration leading to motor and cognitive dysfunction. The CAG (polyglutamine) expansion affects the open reading frame of the HTT gene coding for huntingtin. This leads to pathological deposition of huntingtin protein, and disruption of gene regulation, metabolic, and signaling pathways [193]. Weakened trophic support of neurons and the resulting nuclear accumulation of FOXO3a transcription factor might be an important aspect [194]. The role of sirtuins in neuronal survival and the known interactions with huntingtin [195] and FOXOs [1] made them a plausible research target. However, sirtuins’ role in HD is somewhat controversial, likely stemming from their wide, pleiotropic spectrum of signaling interactions [193]. Till recently, SIRT1 appeared to protect most species from glutamine repeat toxicity, with the notable exception of the Drosophila model [193]. Mutant huntingtin reduces SIRT1 activity, weakening its positive role in neuronal survival. It is possible that the structural similarity between mutant huntingtin and sirtuin-interacting transcription factors might play a role [193]. SIRT1 binds and activates the promoter of brain-derived neurotrophic factor (BDNF); it can also augment the expression of crucial genes such as superoxide dismutase 2, or mitochondrial biogenesis modulators, and can impact Bax signaling via modulation of its binding to Ku70 [196]. However, evidence for neuroprotective influence of selective SIRT1 inhibition in several HD models including mice has been published in recent years; it has been suggested that this approach might augment the clearance of mutant huntingtin [196, 197]. The neuroprotection achieved by SIRT2 inhibition is much more consistent with the current views on its role [193]. The question of possible therapeutic application of sirtuin modulators appears to be tough and highly selective approaches seem necessary.

## Pharmacological Manipulation of Sirtuin Activities for Research and Therapeutic Purposes

A number of pharmacological agents are used to influence the activity of sirtuins for research purposes [198]. HDAC inhibitors display significant level of class specificity: sirtuin inhibitors usually do not affect class I, II or IV enzymes, although the selectivity between sirtuins is a frequent issue [199, 200]. Novel indole compounds seems to offer good specificity and potency while also offering good bioavailability and cell permeability [196]. A new inhibitor 6-chloro-2,3,4,9-tetrahydro-1*H*-carbazole-1-carboxamide (**EX-527**) has been shown to be potent and selective towards SIRT1 [201]. The inhibitor has been used to investigate the role of this isoform in cell physiology and pathology, for example in the regulation of inflammatory responses [202, 203]. In a work on oxidative mitochondrial damage evoked by hyperglycemia the SIRT1 inhibitor has been compared to the effects of siRNA-mediated SIRT1 knock-down [204]. EX-527 has been entered into clinical trials [196]. **AGK2**, an inhibitor selective towards SIRT2 has been used in a study to assess the role of this sirtuin in the toxicity of α-synuclein, mutant huntingtin, and of SIRT2 in cellular energy metabolism [188, 205, 206]. SIRT2 inhibitor **AK-7** was also able to offer neuroprotection in a mouse HD model [193]. 1,2-dihydro-3*H*-naphtho[2,1-*b*]pyran-3-one (splitomicin) [200]; reviewed in [207] has been used as a basis for an array of derivatives with preferential action against SIRT2 versus SIRT1 [208]. The specificity of the widely employed polyphenolic inhibitor 2-[(2-hydroxynaphthalen-1-ylmethylene)amino]-*N*-(1-phenethyl)benzamide (sirtinol) [207] has been recently questioned [209]. 3,4′,5-trihydroxy-trans-stilbene, 5-[(1E)-2-(4-hydroxyphenyl)ethenyl]-1,3-benzenediol (resveratrol), a polyphenol with still unclear mechanism of action has been used to activate sirtuins, with beneficial effects on metabolic regulation, energy metabolism, and organism survival [17, 210]. However, its lack of specificity makes it highly problematic as a research tool [211]. It influences the expression and activity of nitric oxide synthases, catalase, superoxide dismutase, glutathione metabolism, and apoptotic signaling to name a few; only some of these effects are mediated by sirtuins [212]. Despite its shortcomings resveratrol has entered into clinical trials aimed at sirtuins’ role in healthy aging and gender-specific longevity mechanisms, in AD-related cognitive decline, in muscle function in old age, and in the status of a cytoprotective enzyme heme oxygenase-1 [213–216]. Polyphenolic activators of sirtuins also include the powerful and pleiotropic curcumin. The clear need for more specific and selective compounds has led to the identification of a number of new activators such as *N*-(2-(3-(piperazin-1-ylmethyl)imidazo[2,1-b]thiazol-6-yl)phenyl)quinoxaline-2-carboxamide (**SRT1720**), 4-methyl-*N*-[2-[3-(morpholinomethyl)imidazo[2,1-b]thiazol-6-yl]phenyl]-2-(pyridin-3-yl)thiazole-5-carboxamide (**SRT2104**), which has already been shown to protect against neurodegeneration and motor impairment in a mouse HD model [217]. However, despite their therapeutic potential revealed in animal studies and despite some clinical trials on the improvement of the peripheral metabolic health, clinical CNS data are currently lacking [10, 218, 219].

## Conclusion

During the past decade, there has been significant progress in understanding the role of sirtuins in brain aging and in neurodegenerative disorders such as AD [1, 16]. Till now relatively little is known about the role of SIRTs in PD or Huntighton’s disease [5, 196]. The role of SIRT1 in the regulation of APP metabolism and tau deacetylation/phosphorylation should be stressed [147, 148]. SIRT1 expression and activity may significantly affect the course of AD pathology and may be a promising therapeutic target. Recently, studies focused on mitochondrial SIRTs and their roles in antioxidative defense [2]. In oxidative stress and in brain aging/neurodegeration down-regulation of the nuclear SIRT6 may influence DNA repair machinery and probably also telomere maintenance. SIRT6 participates in homologus recombinationation, in non-homologus end-joining, and in base excision DNA repair pathways. It interacts with the transcription factor NF-κB, with PARP and with other proteins engaged in DNA repair; this suggests SIRT6 as another promising target in the regulation of longevity [73, 105]. Till now controversial findings are published on the role of SIRT2 which might be important for longevity but also seems to take part in Aβ production, α-synuclein toxicity, and neuronal cell death [74, 145, 188]. Insufficient data are available on SIRT4 and SIRT5 in mitochondria; the knowledge on sirtuin interactions in the regulation of cell survival and death in physiology and pathology is also leaving something to be desired. Hopefully, further studies will expand our knowledge about application of sirtuin modulators in the therapy of neurodegenerative diseases.
